# Son Preference in Pakistan; A Myth or Reality

**DOI:** 10.12669/pjms.324.9987

**Published:** 2016

**Authors:** Khaula Atif, Muhammad Zia Ullah, Afeera Afsheen, Syed Abid Hassan Naqvi, Zulqarnain Ashraf Raja, Saleem Asif Niazi

**Affiliations:** 1 Dr. Khaula Atif, MBBS, MCPS, DPH, DMA. Department of General Administration, Combined Military Hospital (CMH), Peshawar, Pakistan; 2 Dr. Muhammad Zia Ullah, MBBS, MCPS, MSc. Department of General Administration, Combined Military Hospital (CMH), Peshawar, Pakistan; 3 Dr. Afeera Afsheen, MBBS, MCPS, FCPS. Department of Gynecology. Combined Military Hospital (CMH), Peshawar, Pakistan; 4 Dr. Syed Abid Hassan Naqvi, MBBS, FCPS, FRCS, MS. Department of Ophthalmology, Combined Military Hospital (CMH), Peshawar, Pakistan; 5 Dr. Zulqarnain Ashraf Raja. MBBS, MSc. Department of General Administration, Combined Military Hospital (CMH), Peshawar, Pakistan; 6 Dr. Saleem Asif Niazi, MBBS, MCPS, FCPS, Department of ENT, Combined Military Hospital (CMH), Peshawar, Pakistan

**Keywords:** Under-developed countries, Sex-ratio-at-Birth (SRB), Female Discrimination. Gender Preference

## Abstract

**Objective:**

To analyze desire for sons/daughters among ladies of Peshawar, Pakistan, with a view to rule out son preference and to study impact of various demographic characteristics on the subject.

**Methods:**

Cross-sectional descriptive study conducted at Combined Military Hospital, Peshawar, from August 2015 - January 2016; sampling technique was random/probability/non-purposive. Self-designed questionnaire was utilized; carrying questions pertinent to desire for sons/daughters during marital life, and demographic details. Data analyzed via descriptive analysis (SPSS-21), expressed as frequencies/percentages and mean ± standard deviation(minimum/maximum). Sons and daughters desired (dependent variables) were cross-tabulated with independent variables.

**Results:**

Response rate was 63.25% (n-506). Data revealed following: Sons desired 3.05±2.061(1/12); Daughters desired 1.15±0.767(0/4); 6.1%(n-31) and 0.6%(n-3) desired infinite number of sons and daughters respectively, 18.2%(n-92) did not desire to have even one daughter, while 2.2%(n-11) considered it immaterial to have daughters or sons. There was a significant relation between sons desired and client’s education (p<0.001), husband’s education (p<0.001) and socioeconomic class (p<0.001). There was no significant impact of religion (p-0.142) on desire for sons. Impact of independent variables on daughters desired was similar but less pronounced.

**Conclusion:**

There was candid son preference among the respondents. Gender discrimination can be attenuated by adequately addressing son preference at all tiers.

## INTRODUCTION

Few societies in the world might be unfamiliar with the phrase “Son Preference”; many are facing this dilemma since ages^1^, documented as highest in China, India, Pakistan, Nigeria &North Korea.^2^ Although today’s woman has more liberty in decision making of children to be borne, nevertheless, considerable son preference still keeps them under pressure to bear desired number of sons.^3^ In male dominated societies, gender bias is evident in every field of life, leading to female discrimination since childhood.^1^,^4^ Ladies are given inadequate authority to choose contraceptive practices, criminal female feticide is done, thus leading to altered sex ratio at birth (SRB).^1^,^5^,^6^

In Pakistan, every family must have witnessed gender bias of various degrees. Although such topics have always been the talk of the town, yet, it is minimally investigated/documented. Son preference is a complex issue. It’s the way a parent thinks about her/his off-spring who is not yet born/conceived. In many countries antenatal murder of girls is done, or if born, daughters face the dilemma of discrimination till death. Son preference being so rampant in Pakistan demands to be addressed at grass-root levels.

## METHODS

A cross-sectional descriptive study was carried out in Combined Military Hospital, Peshawar, August 2015 to January 2016); sampling technique was random/probability/non-purposive. Study instrument utilized was a self-designed questionnaire carrying demographic details, questions regarding living off-springs (total children/daughters/sons) and number of sons and daughters desired during marital life. Subjects were married ladies of child-bearing age, from local population of Peshawar, reporting to different OPDs of concerned hospital. Unmarried/post-menopausal ladies and those with stated mental health issues were excluded from the study. Formal approval from the ethical committee of the hospital and written informed consent from participants were taken.

Keeping margin of error 5%, confidence level 95% and response distribution 50%, a sample of 385 individuals was required to represent 3.5 million population of Peshawar. Prior estimation of non-response rate was 40%, therefore, one thousand questionnaires were distributed to volunteers. To ensure privacy and confidentiality, questionnaires were filled in private space of the hospital and only lady doctors assisted the respondents. To make subjects more comfortable, general demographic questions preceded more personal questions related to desire for sons/daughters. Data was analyzed via descriptive analysis (SPSS-21), qualitative data expressed as frequencies/percentages; quantitative as mean ± standard deviation (Minimum/Maximum). Main outcome variables (number of sons/daughters desired) were cross-tabulated with independent variables (age, client’s education, husband’s education, religion and socioeconomic class).

## RESULTS

Response rate was 50.6%(n-506). Mean age(years) 32.3±6.815(22/50); client’s education(years) 6.17±7.168(0/23); husband’s education(years) 9.23±5.990(0/23); number of children 3.46±2.085(0/13); number of daughters 1.42±1.116(0/5); number of sons 2.01±1.581(0/9); children desired 4.85±2.490(2/infinite); daughters desired 1.15±0.767(0/4); sons desired 3.05±2.061(1/12). Infinite number of sons and daughters were desired by 6.1%(n-31) and 0.6%(n-3) individuals respectively; 18.2%(n-92) never desired to have a daughter; while 2.2%(n-11) considered it immaterial to have son or daughter. Break-up of qualitative variables is tabulated (Table-I).

**Table-I:** Frequencies of Qualitative Variables of Study Participants; Frequency/Percentage(n-426).

1 Age Group (Years)	22-29 (190/37.5)	30-39 (219/43.3)	≥40 (97/19.2)	-	-
2 Client’s education	Group-I^1^ (263/52.0)	Group-II^2^ (106/20.9)	Group-III^3^ (90/17.8)	Group-IV^4^ (47/9.3)	-
3 Husband’s education	Group-I^1^ (93/18.4)	Group-II^2^ (203/40.1)	Group-III^3^ (142/28.1)	Group-IV^4^ (68/13.4)	-
4 Religion	Islam (470/92.9)	Christian (36/7.1)	-	-	-
5 Socio-Economic Class	Low (338/66.8)	Middle (146/28.9)	Upper (22/4.3)	-	-
6 Sons Desired	Nil (0/0)	1-2 (260/51.4)	3 or More^5^ (204/40.3)	Infinite (31/6.1)	Immaterial^6^ (11/2.2)
7 Daughters Desired	Nil (92/18.2)	1-2 (380/75.1)	3 or More^5^ (20/4.0)	Infinite (3/0.6)	Immaterial^6^ (11/2.2)

1. Nil

2. Primary to Matriculate

3. Intermediate to Masters

4. Professional/Consultant

5. Finite figure

6. Immaterial to have sons or daughters

Regarding sons desired, there was significant impact of client’s education(p<0.001) and husband’s education(p<0.001), less educated desired for more sons. None of the consultants/professionals wanted to have infinite number of sons, while 90.8% of those who considered it immaterial to have sons or daughters were more educated(45.4% intermediate-masters, 45.4% professional/consultant). Desire for sons was significantly affected by socioeconomic class(p<0.001), economically low ladies had stronger desire to bear more sons.

Similarly, the effects of various demographic characteristics on daughters desired were also significant [client’s education;*p*<0.001, husband’s education;*p*<0.001 and socioeconomic class; *p*<0.001, all were inversely proportional to number of daughters desired. Nevertheless, in all these categories, the desire for daughters was less pronounced than for sons. The comparison between sons and daughters desired through *p*-values and chi-square (of variables having significant *p*-values) is tabulated in Table-II. There was no impact of religion on sons desired(*p*-0.142) or daughters desired(*p*-0.446).

**Table-II:** Cross-tabulation of demographic variables with sons desired & daughters desired with a comparison of *p*-values and chi-square.

Variables	SONS DESIRED					p-value	Chi- Square

	Nil	1-2	≥3^1^	Infinite	Immaterial^2^
1. Client’s Education							
Grade-I^3^	0	95	154	13	1	<0.001	107.412
Grade-II^4^	0	72	24	10	0		
Grade-III^5^	0	55	22	8	5		
Grade-IV^6^	0	38	4	0	5		
Total	0	260	204	31	11		
2. Husband’s Education							
Grade-I^3^	0	20	63	9	1	<0.001	85.195
Grade-II^4^	0	96	87	18	2		
Grade-III^5^	0	90	45	4	3		
Grade-IV^6^	0	54	9	0	5		
Total	0	260	204	31	11		
3. Socio-economic Class							
Low	0	156	158	23	1	<0.001	68.300
Middle	0	94	39	8	5		
Upper	0	10	7	0	5		
Total	0	260	204	31	11		
DAUGHTERS DESIRED							
1. Client’s Education							
Grade-I^3^	61	189	1	11	0	<0.001	53.141
Grade-II^4^	14	87	0	3	2		
Grade-III^5^	5	73	0	6	1		
Grade-IV^6^	12	30	0	0	0		
Total	92	379	1	20	3		
2. Husband’s Education							
Grade-I^3^	29	52	1	11	0	<0.001	56.280
Grade-II^4^	32	159	0	8	2		
Grade-III^5^	25	113	0	0	1		
Grade-IV^6^	6	56	0	1	0		
Total	92	380	1	20	3		
3. Socio-economic Class							
Low	70	251	1	13	2	<0.001	54.599
Middle	19	115	0	6	1		
Upper	3	13	0	1	0		
Total	92	379	1	20	3		

1. Finite figure

2. Immaterial to have sons or daughters

3. Nil

4. Primary to Matriculate

5. Intermediate to Masters

6. Professional/Consultant

## DISCUSSION

**Fig.1:**
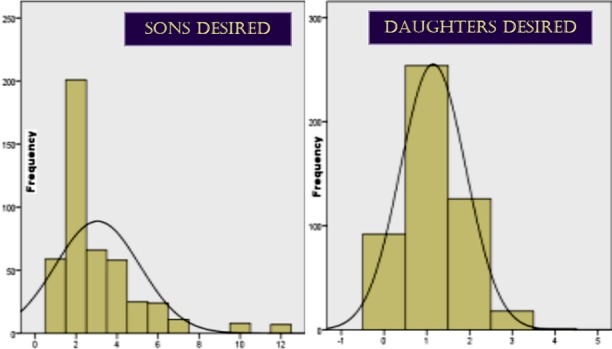
Comparison between number of sons desired & number of daughters desired (n-506).

Many nations depict penchant towards sons; Asia categorically stands out with maximum number of such countries.^5^ In South-Asia, son preference was more pronounced than ever before in last century.^3^,^4^,^6^,^7^ In this study, respondents were found fond of both daughters and sons, but there was a predilection for more sons(Fig.1); validating various local researches.^1^,^8^,^9^ Regional studies documented from of Bangladesh,^6^,^10^ India,^11^-^13^ Srilanka^14^ and Nepal;^15^ stated that none was free of this evil. Although in recent past China has taken dramatic steps to attenuate son preference,^7^ yet SRB is highest there in favor of males(120/100).^16^ In Pakistan, SRB is 1.05(male/female). Internationally, son preference is documented by countries like Malaysia,^17^ Ethopia and Kenya.^18^ In western countries like the United States of America and Canada, although it is doubtful that their natives were free from gender bias, yet they document to owe this evil to immigrants from eastern countries, moreso Asians.^7^,^19^,^20^ South-Asia remains a vital representative of gender bias.^6^,^20^,^21^ adversely affecting women’s basic human rights; nutrition, health care facilities, education etc.^12^ Even contraceptive practices are adopted in accordance with birth of desired number of sons.^3^,^4^,^8^,^10^,^13^,^15^

Apropos, not infrequently, when the “not-so-much-desired” daughter is revealed during antenatal scanning, pregnancy ends up in induced abortions, one after the other, until a “much-more-desired” son is conceived. Female feticide is currently in vogue in many societies; innumerable girls give up their right to live much before their birth; this begin the most brutal form of gender inequality.^5^,^6^,^13^,^16^ In India and China only, this figure crosses millions.^19^ *In civilized nations (USA/Canada), female feticide is documented in* immigrants from China, India, Philippines, Korea And Vietnam.^7^,^19^ Thus the SRB is altered unnaturally;^7^,^12^,^20^ especially in Asia.^5^,^6^ Our data revealed a significantly enhanced desire for infinite number of sons than daughters, every respondent wanted at least one son; while 18.2% never desired a daughter. Researchers reveal that many clients wish to have at least one son,^3^,^11^,^22^ with only one girl if at all born.^11^,^13^

**Fig.2:**
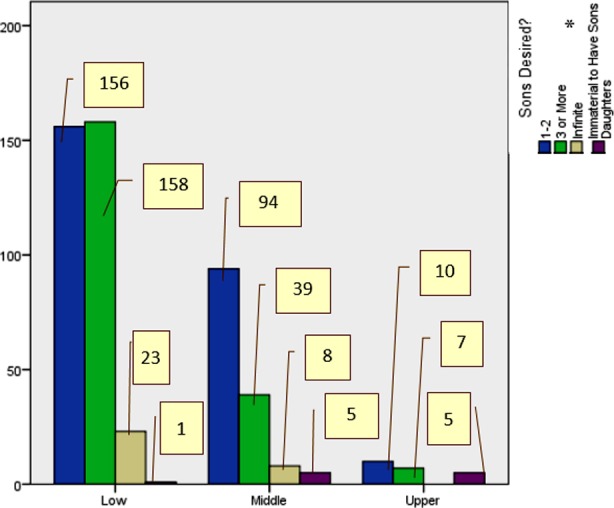
Impact of Socio-Economic Class on Number of Sons desired (n-506). *3 or more means a finite number

This study did not reveal significant predilection for a particular gender of off-springs among Muslims and Christians. Literature revealed similar results.^7^ Regionally, impact of religious beliefs on son preference has been endorsed.^12^ Our data revealed more son preference in low socioeconomic class (Fig.2). Among Asians, particularly in the South-East, son preference is a matter of cultural trend as well socioeconomic dependency on sons; there is a candid bias towards sons especially in lower classes; not for girls as they are not considered socioeconomically as supportive.^1^,^7^,^12^,^21^ Rather, girls are considered liabilities for parents in form of requirement of arranging suitable husbands and hefty dowry.^3^,^21^ In Pakistan, parents of daughters are most worried because they have to arrange suitable marital proposals for them, and a girl’s marriage costs them decades’ savings, while sons become a source of getting dowry on their wedding. Further, there is a lack of social support provided by daughters in old age.^3^ In this study, more educated parents, especially mothers, showed lesser number of sons desired, validated by international literature.^12^,^21^

### Limitations

First, response and recall bias could not be avoided as the questionnaire was self-designed and self-reported. Second, although sample size was big enough to represent the population of Peshawar, but as data was collected from one hospital, therefore, generalization of results can be jeopardized. Third, couples were not interviewed; therefore, the son preference of male partners could not be calculated. Fourth, we did not encompass questions regarding contraceptive practices, family pressures on ladies to bear sons, any marital conflicts/violence or induced abortions. Such data could have rendered excellent results, but adding more variables was beyond the scope of this research.

Despite these short comings, this study was unique and first of its kind as no such study has yet been carried out in Peshawar. It took into account the desired number of sons as well as daughters, and compared both with independent variable. Results unambiguously pointed towards strong son preference. This research may help the policy makers and care takers to address the issue by educating couples to attenuate gender bias. Future studies with better instruments and vaster clientele coverage can be carried out.

## CONCLUSION

Although the respondents showed great penchant for both daughters and sons, nevertheless, latter was much more pronounced. **Perception of parents towards desired gender of off-springs to be borne must be drifted in favor of daughters**, Girls should be given equal chances to be wishfully borne by their parents, and live with equal rights. This **would help in family planning of lesser number of children and would decrease the female discrimination in adulthood as well.**
